# Transcriptomic and metabolomic profiling of long-lived growth hormone releasing hormone knock-out mice: evidence for altered mitochondrial function and amino acid metabolism

**DOI:** 10.18632/aging.102822

**Published:** 2020-02-23

**Authors:** Jessica M. Hoffman, Aliza Poonawalla, Mert Icyuz, William R. Swindell, Landon Wilson, Stephen Barnes, Liou Y. Sun

**Affiliations:** 1 Department of Biology, University of Alabama at Birmingham, Birmingham, AL 35294, USA; 2 Heritage College of Osteopathic Medicine, Ohio University, Athens, OH 45701, USA; 3 The Jewish Hospital, Department of Internal Medicine, Cincinnati, OH 45236, USA; 4 Targeted Metabolomics and Proteomics Laboratory; Department of Pharmacology and Toxicology, University of Alabama at Birmingham, Birmingham, AL 35294, USA

**Keywords:** aging, growth hormone, metabolite, mouse, transcriptomics

## Abstract

Numerous genetic manipulations that extend lifespan in mice have been discovered over the past two decades, the most robust of which has arguably been the down regulation of growth hormone (GH) signaling. However, while decreased GH signaling has been associated with improved health and lifespan, many of the underlying physiological changes and molecular mechanisms associated with GH signaling have yet to be elucidated. To this end, we have completed the first transcriptomic and metabolomic study on long-lived growth hormone releasing hormone knockout (GHRH-KO) and wild-type mice in brown adipose tissue (transcriptomics) and blood serum (metabolomics). We find that GHRH-KO mice have increased transcript levels of mitochondrial and amino acid genes with decreased levels of extracellular matrix genes. Concurrently, mitochondrial metabolites are differentially regulated in GHRH-KO. Furthermore, we find a strong signal of genotype-by-sex interactions, suggesting the sexes have differing physiological responses to GH deficiency. Overall, our results point towards a strong influence of mitochondrial metabolism in GHRH-KO mice which potentially is tightly intertwined with their extended lifespan phenotype.

## INTRODUCTION

The world’s population is aging rapidly, and age is the greatest risk factor for the majority of morbidities that afflict developed nations. In addition, humans are living longer than ever, but they are spending a large proportion of their late life living with multiple chronic conditions. To this end, biologists have been working to develop interventions to delay or slow aging and chronic conditions, leading to increased healthspan as well as lifespan. Over the past several decades numerous dietary, pharmacological, and genetic lifespan extending interventions have been discovered in rodent models. Arguably, the most robust genetic manipulation that leads to repeatable lifespan extension is down regulation of growth hormone (GH) signaling [1, 2]. Ames and Snell dwarf mice are homozygous for mutations, Prop-1 (Ames) and Pit-1 (Snell), that prevents development of cells producing GH, thyrotropin (TSH) and prolactin (PRL) in their anterior pituitary [3–5]. These mutant mice are remarkably long-lived, both in mean and maximum lifespan, and exhibit multiple characteristics that suggest delayed aging. These results have been consistently reproduced in both sexes as well as in animals fed different diets on varying genetic backgrounds [6]. These dwarf mice are the most-studied GH-related mutants in which deficiency of GH is associated with dramatic increases in lifespan. However, in these models, deficiencies of other pituitary hormones may confound direct effects of GH on aging. Mutant mice with only GH deficiency were developed by targeted disruption of the growth hormone releasing hormone (GHRH) gene [7]. Recently, have we shown that these new mutants (GHRH-KO) exhibit lifespan extension robustly in both sexes, as well as major shifts in the expression of genes related to xenobiotic detoxification, stress resistance, and insulin signaling [8]. However, many of the downstream changes and molecular interactions incurred by GH deficiency that might contribute to healthy aging and increased lifespan are unknown.

One approach to elucidate novel pathways that might be dysregulated due to genetic manipulation is through transcriptomics and metabolomics. Transcriptomic and metabolomic analyses have been used to discover novel pathways associated with aging and longevity in multiple model organisms [9–14] and humans [15–17]. These changes are often tissue specific, yet large systemic changes in gene and metabolic regulation can also be seen, leading to the rationale for using different tissues in this study [18]. Moreover, the use of multiple “omics” methods in combination can provide stronger evidence for specific physiological pathways [19].

Here, we complete the first combined transcriptomics and metabolomics analysis of two tissues, brown adipose tissue (transcriptomics) and blood serum (metabolomics), from GHRH-KO mice compared to littermate controls. We aimed to discover changes at the molecular level that occur with GH deficiency as well as novel pathways that are potentially involved in the remarkable longevity extension seen in GH-related mutant mice.

## RESULTS

After data filtering and normalization, our final RNAseq data contained information on 9 (4 males (2 KO, 2 WT), 5 females (2 KO, 3 WT) animals and 14683 individual transcripts. Our final metabolomic dataset consisted of information on 12 animals (3 per group) each with data on 1822 and 1763 metabolites in the positive and negative ion mode, respectively. Basic demographic data on all animals used in the study are shown in Supplementary Table 1, and processed transcriptomics and raw metabolomics data can be found in Supplementary Tables 2–4. Raw transcriptomics data have been submitted to GEO, accession GSE143672.

**Figure 1:**
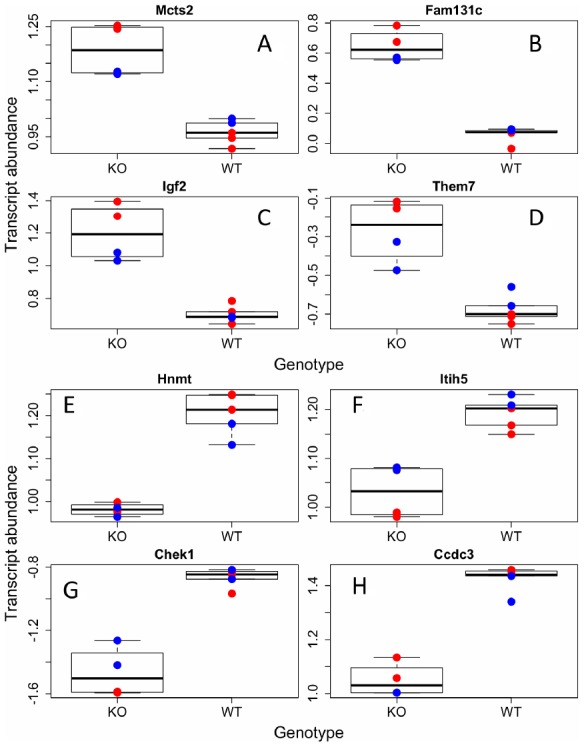
**Individual transcripts differentially regulated in GHRH-KO mouse BAT.** Sample of transcripts that were significantly increased (**A**–**D**) or decreased (**E**–**H**) in KO mutants compared to WT. Red dots indicate females, blue-males.

**Figure 2:**
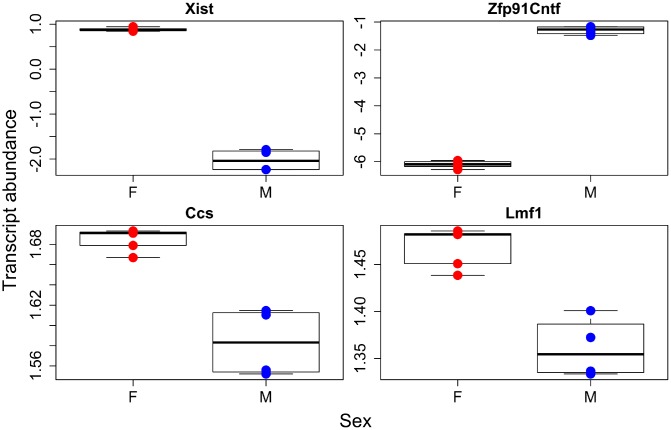
**Individual transcripts with sex effects.** Females in red, males in blue.

We were first interested in identifying genes that were up or down regulated with regards to our factors of interest, sex and GH status. We found 503, 375, and 244 genes were associated with genotype, sex, and their interaction respectively (Supplementary Table 5). We then ran gene ontology on those transcripts that were higher or lower in GHRH-KO mice as compared to WT and higher or lower in males compared to females. We found that KO animals had significantly higher transcript abundances of genes involved in amino acid and mitochondrial metabolism while extracellular matrix and cell division genes were higher in WT animals (Figure 1, Supplementary Table 6). Supplementary Figure 1 depicts a heatmap of those transcripts found to have a significant difference between the two genotypes. Interestingly, we found few sex effects in our ontology analysis, with only two pathways found to be higher in females and none in males (Figure 2, Supplementary Table 6). However, we did find 14 pathways that had genotype by sex interactions; most of these were broadly involved in cofactor and coenzyme metabolism (Supplementary Figure 2, Supplementary Table 6).

**Figure 3:**
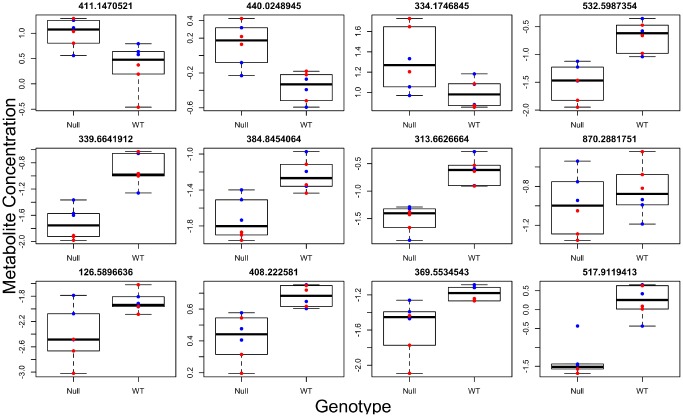
**Individual metabolites changed in GHRH-KO mouse blood serum compared to control mice.** Titles give the mass to charge ratio for each individual metabolite.

**Figure 4:**
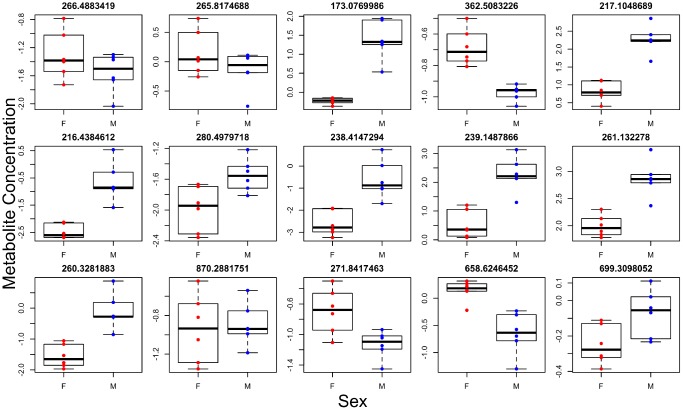
**Individual metabolites with sex effects.** Titles give the mass to charge ratio for each individual metabolite.

Brown adipose tissue (BAT) has systemic effects on metabolism and also has secretory functions leading to the release of factors into circulation [18]. We therefore determined if differences observed in brown adipose tissue (BAT) between the two genotypes were also present systemically. To this end, we ran global metabolomics analysis of blood serum taken from the same animals. We discovered, in the positive and negative ion mode respectively, 33 and 14 individual metabolites associated with genotype (Figure 3, Supplementary Tables 7 and 8), 30 and 28 metabolites associated with sex (most significantly different metabolites shown in Figure 4), and 11 and 8 metabolites with a significant genotype-by-sex interaction at p<0.01. We then ran pathway enrichment on the metabolomic datasets using the program mummichog. We discovered 7 and 2 pathways that were associated with genotype and sex in the positive ion mode (Supplementary Table 9); however, our pathway enrichment analysis failed to find any significant pathways associated with genotype or sex in the negative ion mode. Metabolic pathways found to be associated with genotype were involved in mitochondrial metabolism, specifically response to oxidative stress, as well as serotonin degradation and nicotine degradation. Serotonin degradation metabolism was also found to be different between the sexes, regardless of genotype.

**Figure 5:**
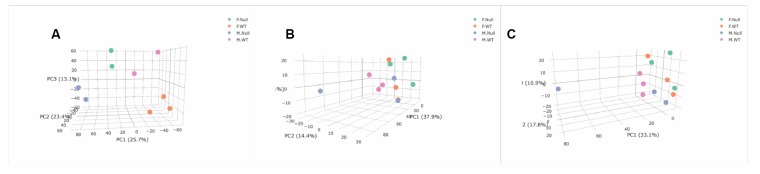
Principal Components Analysis of transcriptome (**A**), positive metabolome (**B**), and negative metabolome (**C**). Female GHRH-KO- green. Female WT- orange. Male GHRH-KO- blue. Male WT- purple. PC3 and PC2 are associated with sex (P=0.0034) and genotype (P=0.0004) respectively in the transcriptome. PC2 was associated with sex in the positive metabolome (P=0.042), and PC3 was associated with sex in the negative metabolome (P=0.017). Genotype was not significantly associated with any of the PCs shown for either metabolomic dataset.

We lastly ran unsupervised Principal Components Analysis (PCA) on both the transcriptomic and metabolomic datasets to determine if either the entire transcriptomes or metabolome were associated with genotype and sex. The BAT transcriptome showed separation of all four groups (Figure 5A). However, in the blood metabolome, sex appears to be a stronger factor delineating the groups with female KO and WT mice more similar to each other than to either group of males (Figure 5B). In the positive ion mode, both genotypes of female mice and GHRH-KO male mice showed similar metabolomic profiles, and only the KO males had different profiles than the other groups (Figure 5C). However, some of these results are due to a strong male KO animal that was an outlier compared to all other individuals. Overall, our PCA results suggest that the BAT transcriptome shows more differences in both genotype and sex than our blood metabolomic analysis, similar to what was seen with individual transcripts and metabolites.

## DISCUSSION

Here we have presented one of the first studies to understand the genetic regulatory and metabolic changes that occur in adult mice with GH mutation, and the first in GHRH-KO mice. Overall, our results show striking differences in individual transcript levels between WT and GHRH-KO mice, genes related to mitochondrial function were significantly upregulated in knockout mice compared to controls. These results suggest that mitochondrial function is altered in the BAT of the GHRH-KO animals, which is not surprising as GH related mutant mice have reported increases in total BAT mass per body weight with higher metabolic activity [6, 20, 21]. Previous research suggests that GH disruption can lead to significant changes in mitochondrial metabolism [reviewed in 22]. GHR-KO (Growth hormone receptor knockout) mice show increased levels of genes related to mitochondrial biogenesis across tissues [23]. In a similar vein, Snell dwarf mice show improved mitochondrial function in response to doxycycline induced oxidative stress [24]. In addition, our metabolomic analysis also suggests mitochondrial function pathways were significantly different between GHRH-KO and WT mice, suggesting that the transcriptomic differences seen in BAT are also observed systemically throughout the animal.

Interestingly, we found significant upregulation of insulin like growth factor 2 (IGF-2) in GHRH-KO mice compared to controls (Figure 1), not a downregulation of IGF-I, as expected. IGF-I was higher in WT animals; however, it did not pass our alpha (p=0.02). Decreasing GH action significantly reduces levels of IGF-I *in vivo*, but the effects of GH on IGF-2 in mice are not completely known. IGF-2 is a major developmental growth regulator and has not been shown to change much in adulthood, unlike IGF-I that is upregulated from development to adulthood, and then declines with older age [25]. Our results indicate IGF-2 transcription is upregulated in GH disrupted mice. As IGF-2 has been largely ignored in the aging field, even with its homology and similar functions to IGF-I, it will be interesting to note in future studies if increases in IGF-2 are associated with health and longevity.

We also discovered an upregulation of amino acid metabolism genes in GHRH-KO animals suggesting that they may have the ability to break down amino acids more effectively than WT mice. Potentially, GHRH-KO mice are able to break down nutrients, specifically amino acids, better than WT mice; thus, leading to better utility of nutrients. It has been well documented that decreases in nutrient acquisition and use lead to increases in lifespan, through either calorie restriction or reduction in amino acids [26], and potentially GH mutant mice are working though similar mechanisms. In addition, breakdown of amino acids has been implicated in energy processes. Higher levels of amino acid metabolism genes in GHRH-KO mice may also indicate they are able to immediately metabolize nutrients such that the negative effects of amino acid excess are readily removed from the system, and previous research suggests that GH can lead to an increase in amino acid uptake [27]. Thus, GH mutant mice may uptake fewer amino acids and catabolize those they do uptake more efficiently. While our transcriptomic analysis points towards a strong role in nutrient metabolism, our metabolomic analysis failed to recapitulate these differences in amino acid profiles systemically in the blood; however, several individual amino acids (valine and proline), which perhaps counterintuitively, were found to be increased in GHRH-KO mice as compared to WT.

Our sex specific analysis failed to find many transcript pathways that were either higher in females or males. However, as a proof of principal the most significantly different transcribed gene between the sexes was *Xist*, the X inactive specific transcript. This gene causes one copy of the X chromosome to become inactivated, and as females have two copies of the X, it would be expected they should have significantly higher levels of *Xist*. We do see this difference, which leads overall credence to our transcriptome analysis. We found over 300 transcripts that were differentially expressed between males and females, and this combined with the lack of gene ontology results, suggests that the sex differences in transcription appear to be broad across all of metabolism not just enriched for a small number of metabolic processes. Along the same lines, our metabolomic analysis found significant differences in the sexes, but We failed to discover much pathway enrichment. The metabolome and transcriptome of the sexes are definitely different as evident by the separations seen in our PCA for all datasets, but these differences are not confined to specific pathways but instead are more diffuse across all physiological processes.

While we find striking differences between GHRH KO mice and WT individuals, our study is not without caveats. Given the large numbers of metabolites and transcripts analyzed for the metabolomic study, we have a relative limited sample size study. However, we find the consistent differences in individual metabolites and transcripts suggesting that there are many interesting differences in the GHRH-KO mice. Adding additional support to our study, our Principle Components Analysis of both the metabolome and transcriptome suggests the physiology of WT and KO animals are significantly different.

Another limitation is with annotation of our metabolomics data. As global metabolomics only provides a mass to charge ratio and retention time for each analyte, annotation software is required to determine an identity for each individual metabolite. However, we were only able to annotate a small proportion of the metabolites in the study (~15%), and misannotation is potentially a large issue. One of the major limitations that still needs to be overcome in metabolomics is improvements in annotation abilities for global, untargeted studies.

## CONCLUSIONS

Here, we have presented one of the first metabolome and transcriptome studies in GHRH-KO mice. Our results suggest potential upregulation of mitochondrial and amino acid metabolism in BAT in animals with down regulated GH signaling, and our metabolomic analysis hints at systemic changes in blood serum with regards to mitochondrial and amino acid metabolism as well. Future studies will investigate if perturbations of these individual metabolites and genes in WT mice can cause similar improvements in physiological similar to GHRH-KO mice. Overall, our results indicate several novel IGF-I independent pathways that may contribute to lifespan extension in GH mutant mice.

## MATERIALS AND METHODS

### Mouse husbandry and sample collection

GHRH-KO mice and their littermate controls (on a mixed C57BL6 and 129SV background) were bred in the colony derived from Roberto Salvatori laboratory [7] and housed under standard conditions (12-hr light/12-hr dark cycle at 20–23°C). Animals had access to *ad libitum* water and Lab Diet Formula 5001 mouse chow (23% protein, 4.5% fat, 6% fiber, Nestle Purina, St. Louis, MO). Demographics of animals used in the study are shown in Supplementary Table 1. Mice were anaesthetized with Isoflurane and cervical dislocation was performed. Whole blood was collected via cardiac puncture and immediately placed on ice in a 1.5-mL centrifuge tube for 30 minutes and then spun at 10,000 rpm for 10 minutes. Serum was then transferred to a fresh 1.5-mL centrifuge tube and stored at −80°C. Brown adipose tissue (BAT) and other tissues were immediately harvested, snap-frozen in liquid nitrogen and then stored at −80°C. Animal protocols were approved by the Animal Care and Use Committee of UAB and SIUSOM.

All statistical analyses were completed in the language R unless otherwise stated [28], and for metabolomics analysis, all analyses were run on both the positive and negative ion data individually.

### RNA sequencing and data analyses

RNA sequencing was performed on Illumina HiSeq2000 platform with 2×50bp paired-end sequencing configuration. Briefly, the quality of the total RNA was assessed using the Agilent 2100 Bioanalyzer followed by 2 rounds of poly A+ selection and conversion to cDNA. The TruSeq library generation kit was used to construct the cDNA library as per the manufacturer’s instructions (Illumina, San Diego, CA). The cDNA library was quantitated using qPCR in a Roche LightCycler 480 with the Kapa Biosystems kit for library quantitation (Kapa Biosystems, Woburn, MA) prior to sequencing. Paired end 2 × 50 bp sequencing runs were performed in the Illumina HiSeq2500. Sequence data were converted to FASTQ Sanger format using Illumina's bcl2fastq version 1.8.4 and aligned to the University of California, Santa Cruz mouse mm10 genome using TopHat version 2.0.11 and the short-read aligner Bowtie was used to assemble transcripts and estimate abundances.

In the study, a transcript was removed from the study if any sample had a FPKM (fragments per kilobase million) of zero. First, transcript FPKM values were log transformed to attain normality. We were first interested in determining those individual transcripts that were significantly different between GHRH and control mice, controlling for the effects of sex. Therefore, we ran a linear model on the effects of sex, genotype, and their interaction on transcript levels, setting significance for any factor as P<0.01. A heatmap was constructed of those metabolites that were significantly associated with genotype. We then ran Gene Ontology analysis on significant transcripts using the *goseq* package in R [29]. Pathways were considered to be significantly enriched at a false discovery rate (FDR) <0.05. Finally, we ran a Principle Components Analysis (PCA) on transcript data to determine if the entire transcriptome was associated with genotype and sex. For this specific analysis, data was centered and scaled to a mean of 0, standard deviation of 1 to meet the assumptions of PCA. 3D PCA figures were created with the *plotly* package [30].

### Metabolomics and data analysis

200μL of serum was added to 750μL of 1:2 (v/v) of chloroform:methanol and vortexed thoroughly. Next, 250μL of chloroform was added, followed by mild vortexing. Finally, 250μL of ddH_2_0 was added to each sample and vortexed. Samples were then vortexed at 1000rpm at room temperature for 5 min to separate layers. The top layer was removed and placed in a micro centrifuge tube, and this new sample was then evaporated using a speed vacuum. 500μL of ice cold 80% Methanol was added to dried samples for 30 minutes and then centrifuged at 14000rpm to precipitate additional protein content. Final supernatant was collected into a new microcentrifuge tube and evaporated. 100μL of 0.1% Formic Acid in ddH_2_0 for mass spectrometry evaluations.

An aliquot (5 μL) of each extracted sample was loaded onto a Nano cHiPLC 200μm x 6mm ChromXP C18-CL 3μm 120Å reverse-phase trap cartridge (Eksigent, Toronto, Canada) at 2 μL /min using an Eksigent autosampler. After washing the cartridge for 5 min with 0.1% formic acid in ddH_2_0, the bound peptides were flushed onto a Nano cHiPLC column 200μm x 15cm ChromXP C18-CL 3μm 120Å (Eksigent, Toronto, Canada) with a 20 min linear (2-98%) acetonitrile gradient in 0.1% formic acid at 1000 nl/min using an Eksigent 400 NanoLC System. (Dublin, CA). The column was washed with 98% acetonitrile-0.1% formic acid for 5 min and then re-equilibrated with 2% acetonitrile-0.1% formic acid for 5 min. A SCIEX 5600 Triple-time of flight mass spectrometer (SCIEX, Toronto, Canada) was used to analyze the protein digest. The IonSpray voltage was 2300 V and the declustering potential was 80 V. Ionspray and curtain gases were set at 10 psi and 25 psi, respectively. The interface heater temperature was 120°C. Eluted metabolites were subjected to a time-of-flight 250msec survey scan from 50-1000 m/z to determine the top twenty most intense ions for MS/MS analysis. Product ion time-of-flight scans at 50 msec were carried out to obtain the tandem mass spectra of the selected parent ions over the range from *m/z* 50-1000 using a rolling collision energy parameter to determine the best fragmentation energies per compound mass. Final LC-MS data were processed using XCMS-Online [31] to identify and align peaks occurring across all samples.

Metabolite concentrations were log-transformed, and values in each sample were centered and scaled to a mean of zero and a standard deviation of one. Metabolites that were not found in all samples were removed from analysis. Similar to RNAseq analysis, we ran a basic linear model looking at the effects of genotype, sex, and their interaction on metabolite concentration. Metabolites were considered to be associated with a factor of interest at p<0.01. For those metabolites that were found to be significantly different between groups, we attempted to annotate them with a putative identity using mummichog [32]. We were then interested in determining if the entire metabolome was associated with the genetic background of the animals by running unsupervised PCA using the same methods as described in the RNAseq analysis.

## Supplementary Material

Supplementary Figures

Supplementary Tables

Supplementary Table 2

Supplementary Table 3

Supplementary Table 4

Supplementary Table 5

Supplementary Table 6

Supplementary Table 7

Supplementary Table 8
