# Supplementary material for: Transcriptomic and metabolomic profiling of long-lived growth hormone releasing hormone knock-out mice: evidence for altered mitochondrial function and amino acid metabolism
Source: Aging (Albany NY). 2020 Feb 23;12(4):3473–85. doi: 10.18632/aging.102822 (PMC7066919; doi:10.18632/aging.102822)
Supplement: Supplementary Figures [file aging-12-102822-s007..pdf]

SUPPLEMENTARY FIGURES

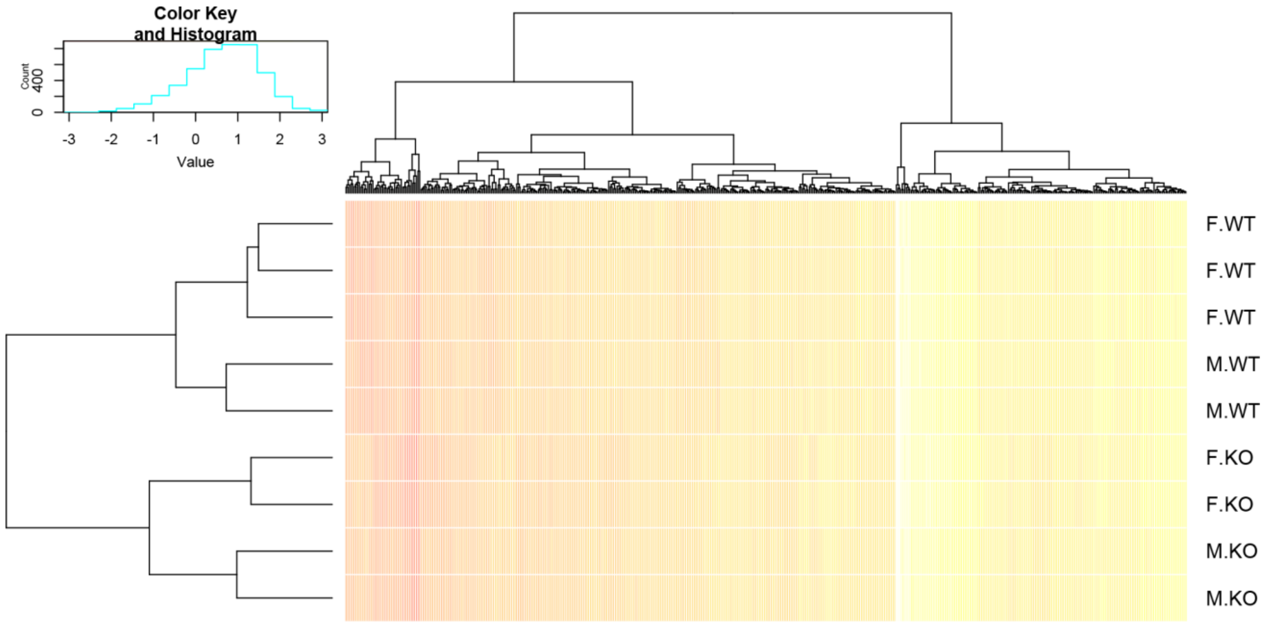

Supplementary Figure 1. Heatmap of transcripts associated with genotype.

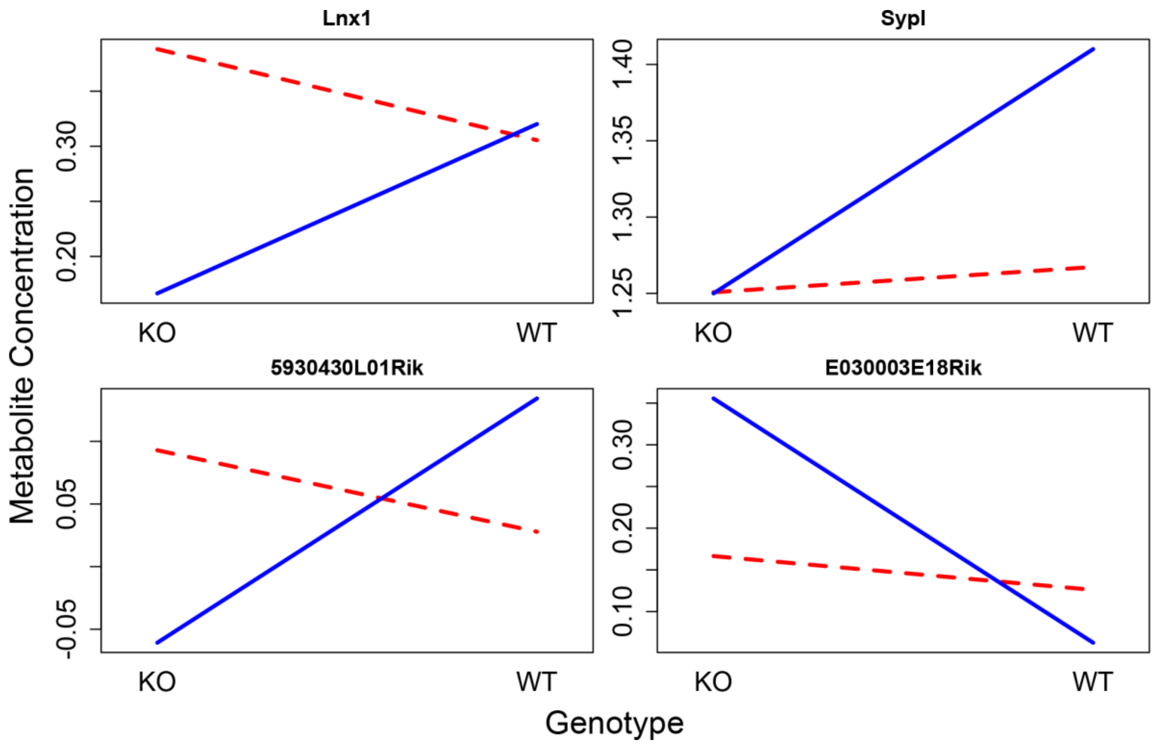

Supplementary Figure 2. Individual transcripts with genotype by sex interactions. Blue, solid lines indicate males while red, dotted lines indicate mean female values.
