# Supplementary material for: Transcriptomic and metabolomic profiling of long-lived growth hormone releasing hormone knock-out mice: evidence for altered mitochondrial function and amino acid metabolism
Source: Aging (Albany NY). 2020 Feb 23;12(4):3473–85. doi: 10.18632/aging.102822 (PMC7066919; doi:10.18632/aging.102822)
Supplement: Supplementary Tables [file aging-12-102822-s006..pdf]

SUPPLEMENTARY TABLES

Supplementary Table 1. Mouse demographic information.

Mouse ID	Genotype	Sex	Weight	Transcriptomics	Metabolomic
10722	Null	M	14.01	x	x
10723	Null	M	13.46	x	x
10724	Null	M	13.4		x
10727	WT	M	26.22	x	x
10728	WT	M	27.34		x
10729	WT	M	25.99	x	x
10713	WT	F	27.63	x	x
10714	WT	F	27.03	x	x
10715	WT	F	26.44	x	x
10717	Null	F	12.07	x	x
10718	Null	F	13.96		x
10720	Null	F	12.31	x	x

Please browse Full Text version to see the data of Supplementary Tables 2–8

Supplementary Table 2. FPKM transcriptomic data.

Supplementary Table 3. Raw positive ion mode metabolomics data.

Supplementary Table 4. Raw negative ion mode metabolomics data.

Supplementary Table 5. RNAseq linear model pvalues.

Supplementary Table 6. Gene ontology pathways up or down regulated in GHRH-KO mice. Note GHRH-KO mice have elevated gene transcript abundances of mitochondria and amino acid metabolism genes.

Supplementary Table 7. Positive ionization mode metabolite liinear model pvalues.

Supplementary Table 8. Negative ionization mode metabolite linear model pvalues.

Supplementary Table 9. Significantly enriched metabolic pathways in blood serum.

Variable-Ionization mode	Pathway	Significnatly different metabolites	Metabolites in pathway	Adjusted p-value
Genotype-Positive				
	Nicotine degradation III	4	8	0.014
	Serotonin degradation	3	5	0.018
	Ceramide biosynthesis	2	2	0.027
	Mineralocorticoid biosynthesis	2	2	0.027
	Glutathione redox reactions I	2	2	0.027
	Glutathione redox reactions II	2	2	0.027
	Nicotine degradation IV	3	7	0.042
Sex-Positive				
	CMP-*N*-acetylneuraminate biosynthesis I	2	3	0.007
	Serotonin degradation	2	4	0.009

Differentially regulated metabolic pathways for genotype and sex were identified by the program mummichog. Enriched pathways were only discovered in the positive ionization mode.
